# 

**Published:** 1898-09-03

**Authors:** 


					THE HOSPITAL. "The standard of highest purity." ?Lancet, Sept. 3rd, 1898.
CADBURY'S YJf te*? CADBURY'S C
COOOA *1 COCOA
ABSOLUTELY PURE, __ J NO DRUGS OR ALKALIES USED.
iRWICH HOSPITAL
?    yLMati"~ r- S J fcH/V Infihmaht
No. 623. Vol. XXIV. USSSJ5] Saturday,
Sept. 3rd, 1898,
[Sntaaripton Tern.,] pRICE TWOPENCE.

				

